# Improved adhesive properties of recombinant bifidobacteria expressing the *Bifidobacterium bifidum*-specific lipoprotein BopA

**DOI:** 10.1186/1475-2859-11-80

**Published:** 2012-06-13

**Authors:** Marita Gleinser, Verena Grimm, Daria Zhurina, Jing Yuan, Christian U Riedel

**Affiliations:** 1 Institute of Microbiology and Biotechnology, University of Ulm, 89069, Ulm, Germany; 2 Institute of Disease Control and Prevention, Academy of Military Medical Sciences, Beijing, China

**Keywords:** Adhesion, Bifidobacteria, Intestinal epithelial cells, Probiotics

## Abstract

**Background:**

Bifidobacteria belong to one of the predominant bacterial groups in the intestinal microbiota of infants and adults. Several beneficial effects on the health status of their human hosts have been demonstrated making bifidobacteria interesting candidates for probiotic applications. Adhesion of probiotics to the intestinal epithelium is discussed as a prerequisite for colonisation of and persistence in the gastrointestinal tract.

**Results:**

In the present study, 15 different strains of bifidobacteria were tested for adhesion. *B. bifidum* was identified as the species showing highest adhesion to all tested intestinal epithelial cell (IEC) lines. Adhesion of *B. bifidum* S17 to IECs was strongly reduced after treatment of bacteria with pronase. These results strongly indicate that a proteinaceous cell surface component mediates adhesion of *B. bifidum* S17 to IECs. *In silico* analysis of the currently accessible *Bifidobacterium* genomes identified *bopA* encoding a lipoprotein as a *B. bifidum*-specific gene previously shown to function as an adhesin of *B. bifidum* MIMBb75. The *in silico* results were confirmed by Southern Blot analysis. Furthermore, Northern Blot analysis demonstrated that *bopA* is expressed in all *B. bifidum* strains tested under conditions used to cultivate bacteria for adhesion assays. The BopA gene was successfully expressed in *E. coli* and purified by Ni-NTA affinity chromatography as a C-terminal His_6_-fusion. Purified BopA had an inhibitory effect on adhesion of *B. bifidum* S17 to IECs. Moreover, *bopA* was successfully expressed in *B. bifidum* S17 and *B. longum/infantis* E18. Strains overexpressing *bopA* showed enhanced adhesion to IECs, clearly demonstrating a role of BopA in adhesion of *B. bifidum* strains.

**Conclusions:**

BopA was identified as a *B. bifidum*-specific protein involved in adhesion to IECs. *Bifidobacterium* strains expressing *bopA* show enhanced adhesion. Our results represent the first report on recombinant bifidobacteria with improved adhesive properties.

## Background

Bifidobacteria are Gram-positive bacteria that represent one of the major genera of the intestinal tract of humans and animals [1]. Several beneficial effects on the health status of the human host have been claimed to be related to the presence of bifidobacteria in the colon (reviewed in [2-4]), thus they become increasingly interesting for probiotic applications in pharmaceutical and dairy products. Several criteria are used for the selection of probiotic strains. Besides manufacturing criteria and shelf life, species- and strain-specific properties related to the probiotic effects are of major importance [5,6].

One of the most commonly tested parameters is the ability of a probiotic to colonise the gastrointestinal tract (GIT) of the host. This is associated with resistance to the conditions of the GIT (low pH, high concentration of bile salts) and adhesion to mucus and/or IECs. While there are a number of studies assessing the adhesion of probiotic bacteria quantitatively [7-10] to date only very few have investigated adhesion of these organisms from a mechanistic point of view. Most mechanistic studies on adhesion to IECs were performed for pathogenic bacteria such as enteropathogenic *E. coli* (EPEC), *Salmonella* sp., streptococci, and *Listeria monocytogenes*[11-14].

Nevertheless, a few authors have reported on the mechanisms of adhesion of probiotic bacteria. Since a number of tools for the genetic modification of lactobacilli are available these organisms have been the subjects of most of these studies [15]. By contrast, very little is known on the mechanisms of bifidobacterial adhesion to IECs. Adhesion of *B. breve* strain 4 to IECs is mediated by a proteinaceous component present on the cell surface and in spent culture supernatant [16]. Binding of human plasminogen *in vitro* was shown for four bifidobacterial strains belonging to three different species (*B. lactisB. bifidum* and *B. longum*) and this process is believed to play a role in the interaction with host tissues [17-19]. A cell surface lipoprotein was shown to be involved in adhesion of *B. bifidum* MIMBb75 to IECs [20]. Moreover, adhesion of this strain to Caco-2 and HT29 cells was dependent on environmental conditions, such as pH and the presence of sugars and bile salts [21].

However, while adhesion might play an important role in establishing administered probiotic bacteria in the intestinal tract of the host, to date no direct correlation between the health-promoting properties of probiotics and their adhesion to IECs and/or mucus could be shown. Recently, our group could describe for the first time a correlation between adhesion of bifidobacteria to IECs and their anti-inflammatory capacity *in vitro*. Bifidobacteria showed strain- and species-specific adhesion to Caco-2 and T84 cells [22,23]. Furthermore, the inhibition of LPS-induced NF-κB activation, pro-inflammatory gene expression and IL-8 secretion in IECs by bifidobacteria *in vitro* is strain- and dose-dependent [22,24]. Interestingly, those strains that showed high levels of adhesion were also those that performed best in inhibiting LPS-induced NF-κB activation [22]. Two strains with opposing characteristics were thus further tested in different murine models of intestinal inflammation. In all models, animals treated with *B. bifidum* S17, a highly adherent strain, were protected from weight loss, had a normalised colonic weight to length ratio and showed improved histological scores. By contrast, the weakly adherent *B. longum*/*infantis* E18 had no protective effect [22,25].

Based on these results, the strain *B. bifidum* S17 was selected to investigate its adhesive properties aiming to identify the components involved in adhesion to T84, Caco-2 and HT29 cells and to improve the adhesive properties of this and other bifidobacterial strains.

## Results

### Adhesion of different *Bifidobacterium* strains to IECs

**Figure 1:**
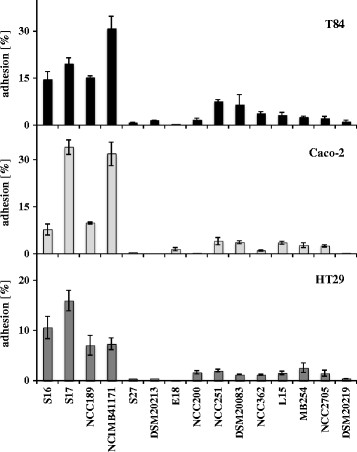
**Adhesion of different*Bifidobacterium*strains to IECs**. Adhesion of *B. bifidum* strains S16, S17, NCC189 and NCIMB41171, *B. breve* strains S27 and DSM20213, *B. longum/infantis* strains E18 and NCC200, *B. adolescentis* strains NCC251 and DSM20083, *B. lactis* strains NCC362 and L15, *B. animalis* MB254, and *B. longum* strains NCC2705 and DSM20219 to T84 (black bars), Caco-2 (light grey bars) and HT29 (dark grey bars) cells. Adherent bacteria were determined as colony forming units on MRSC agar plates and expressed as percentage of the initially added bacteria (10^8^ cfu/well). Values are mean ± standard deviation of one representative of at least three independent experiments performed in triplicate measurements.

In previous studies, different bifidobacteria were shown to adhere in a strain-specific manner to T84, Caco-2 and HT29 cells [22,23]. The results indicated that especially strains of *B. bifidum* showed high adhesion to IECs. However, only a limited number of strains were tested. Thus, adhesion to different cell lines of IECs was tested with an extended range of strains including four *B. bifidum* strains. In line with previous observations, bifidobacteria adhered to all tested IEC lines in a strain-specific manner. In comparison to all other *Bifidobacterium* strains tested, *B. bifidum* strains showed significantly higher levels of adhesion to IECs, with adhesion rates of up to 30% depending on the cell line used (Figure 1; Additional file 1: Table SA 1). Of note, all of the tested strains showed lower adhesion to HT29 compared to T84 and Caco-2 cells.

We and others have recently sequenced and published the genomes of different strains of *B. bifidum*[26,27]. Based on *in silico* analysis, several proteins with domains known or supposed to be involved in interaction with host tissue were identified in the genome sequence of *B. bifidum* S17 (data not shown). The identified proteins include a protein with high homology to the bifidobacterial outer protein A (BopA), which has been described as a lipoprotein associated with the cell wall of *B. bifidum* MIMBb75 and was shown to be involved in adhesion to cultured IECs [20]. Since *B. bifidum* strains showed higher adhesion to IECs than all other strains tested, we hypothesised that BopA is a unique feature of the *B. bifidum* strains involved in the strong adhesion of this species to IECs.

### Homologies between BopA-coding loci of different *B. bifidum* strains

In order to identify possible homologues of BopA in bifidobacteria, the sequence of *bopA* was compared to all bifidobacterial genome sequences publically available on the NCBI web site using BLAST analysis. The analysis covered 32 genomes including the following species: *B. bifidum* (strains S17, PRL2010, and NCIMB41171), *B. longum* ssp. *longum* (strains NCC2705, DJO10A, BBMN68, F8, JCM1217, JDM301, and KACC 91563), *B. longum* ssp. *infantis* (strains 157 F and ATCC 15697), *B. breve* (strains UCC2003, DSM20213, and ACS-071-V-Sch8b), *B. adolescentis* (strains ATCC15703 and L2-32), *B. dentium* (strains Bd1, ATCC 27678, ATCC 27679, and JCVIHMP022), *B. animalis* ssp. *lactis* (strains AD011, BB-12, BLC1, Bl-04, DSM10140, and V9), *B. pseudocatenulatum* DSM20438, *B. catenulatum* DSM16992, *B. angulatum* DSM20098 and *B. gallicum* DSM20093. The results indicated that BopA is indeed present only in *B. bifidum* strains since in no other genome a protein with significant homology to BopA was found (Additional file 2: Table SA 2).

**Figure 2:**
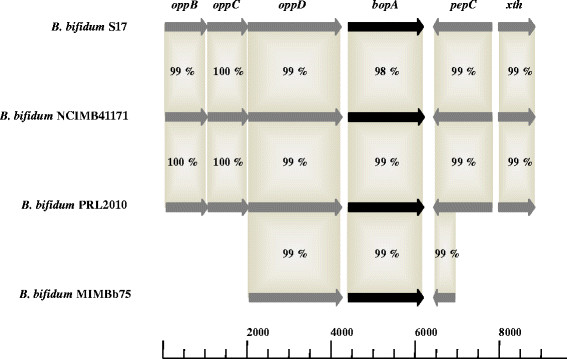
**Homologies between BopA regions of*B. bifidum*strains.** Comparative *in silico* analysis of the genomic loci of *B. bifidum* S17, NCIMB41171, PRL2010 and MIMBb75 encoding *bopA* and adjacent genes by BLAST analysis. Open reading frames (ORFs) are indicated as arrows sized proportional to the length of the genes (length in basepairs is indicated in the scale). Identities of the deduced amino acid sequences compared to the *B. bifidum* S17 protein are indicated in percent. For *B. bifidum* MIMBb75 no sequence information is available for the regions upstream of *oppD* and downstream of *pepC*.

The locus encoding *bopA* was further analysed by comparing the respective loci of those *B. bifidum* strains for which sequence data was publically available. These strains included *B. bifidum* S17, NCIMB41171, PRL2010 and MIMBb75 (NCBI accession numbers: CP002220, NZ_ABQP00000000, CP001840, AM710395; Figure 2). This analysis revealed that *bopA* is part of a putative peptide-specific ATP-binding cassette (ABC) transport system with similarity to oligopeptide permease (Opp) of other bacteria. In *Salmonella typhimurium* the transporter is encoded by the *oppABCDF* operon, in which OppA represents the solute binding protein, OppB and OppC are two permeases that span the cytoplasmic membrane, and OppD and OppF are the ATP-binding and ATP-hydrolysing proteins [28,29]. *B. subtilis* possesses a functional equivalent of the Opp peptide transporter, encoded by the operon *oppABCDF*, which is similar in sequence and organisation to that of Gram-negative bacteria [30,31]. However, the *Bacillus* OppA is linked to the cytoplasma membrane via a lipid anchor and it has been shown OppF is not essential for functionality of the transporter [31,32].

The analysis of bifidobacterial genomes revealed that various species sequenced so far harbour genes with similarity to the *opp* genes of *B. subtilis* and *S. typhimurium* (data not shown). In *B. bifidum* strains the *oppA* homologue was termed *bopA*[20]. Moreover, instead of *oppF*, a *pepC* gene coding for an aminopeptidase C has been identified in the *opp* locus of *B. bifidum* strains downstream of *bopA* on the opposite strand (Figure 2; Additional file 3: Table SA 3). The BopA protein of *B. bifidum* MIMBb75 is annotated as putative solute binding protein and was shown to harbour a potential thioacylation site for covalent linkage to a conserved cysteine residue in the lipobox motif [20].

Comparative *in silico* analysis of the *bopA* region of various *B. bifidum* strains showed that the *bopA* gene is highly conserved in this species (Figure 2 and additional File 4: Figure S 1). The deduced amino acid sequence of BopA from *B. bifidum* S17 shows 98% identity with BopA of *B. bifidum* NCIMB41171 and 99% identity with BopA of *B. bifidum* PRL2010 and *B. bifidum* MIMBb75, respectively (Figure 2). Furthermore, all sequences harbour the conserved cysteine residue in the lipobox motif (Additional file 4: Figure S 1).

### Detection and expression analysis of *bopA* in *B. bifidum* strains

**Figure 3:**
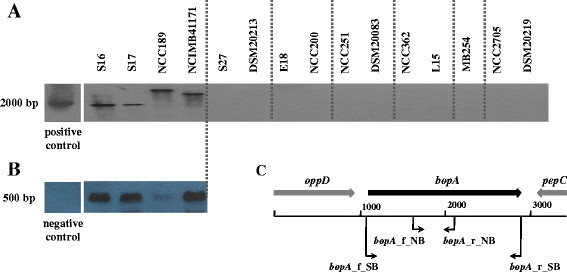
**Detection of*bopA*in*B. bifidum*strains and expression analysis.** (**A**) Southern Blot analysis for *bopA* homologues using chromosomal DNA of *B. bifidum* strains S16, S17, NCC189 and NCIMB41171, *B. breve* strains S27 and DSM20213, *B. longum/infantis* strains E18 and NCC200, *B. adolescentis* strains NCC251 and DSM20083, *B. lactis* strains NCC362 and L15, *B. animalis* MB254, and *B. longum* strains NCC2705 and DSM20219. As positive control for hybridisation, a non-labelled PCR product of *bopA* of *B. bifidum* S17 was used. (**B**) Expression analysis of *bopA* was performed by Northern Blot using RNA isolated from *B. bifidum* strains S16, S17, NCC189 and NCIMB41171 after growth for 16 h in MRSC medium. As negative control RNA isolated from *B. longum/infantis* E18 was used. (**C**) Schematic representation of the *bopA* locus with localisation of oligonucleotides used for amplification of the probe for Southern Blot (1,812 bp) and Northern Blot (489 bp).

To confirm the results of the *in silico* analysis, Southern Blot analysis was performed on genomic DNA of all *Bifidobacterium* strains tested for adhesion (Figure 3A). For this purpose, genomic DNA of all strains was digested with *Eco*RI since *in silico* analysis indicated that the *bopA* genes of the sequenced strains do not harbour an *Eco*RI restriction site. When digested DNA was probed for the presence of *bopA* homologues using a digoxygenin-labelled 1,812 bp probe covering the entire *bopA* gene (Figure 3C) a clear signal was detected for all four tested *B. bifidum* strains (S16, S17, NCC189 and NCIMB41171; Figure 3A). By contrast, the probe did not hybridise to genomic DNA of any of the other strains tested indicating that these strains do not harbour a gene with homology to *bopA*.

In order to verify whether *bopA* is expressed, Northern Blot analysis was performed for the four *B. bifidum* strains (S16, S17, NCC189 and NCIMB41171) using RNA isolated from bacteria grown under conditions used for adhesion assays. Using a *bopA*-specific digoxygenin-labelled probe (Figure 3C) a signal could be detected for all four *B. bifidum* strains tested (Figure 3B). However, signal intensities were variable amongst the strains. Nevertheless, these results demonstrate that *bopA* is expressed in these strains after growth in MRSC medium for 16 h under standard conditions. Since bifidobacteria were cultivated under these conditions to perform adhesion assays it is assumed that BopA is present on the surface of these strains when used for adhesion assays. In summary, the results of these experiments suggest that BopA is a *B. bifidum*-specific lipoprotein with a role in the strong adhesion of *B. bifidum* strains to IECs.

### Adhesion of *B. bifidum* S17 is mediated by a cell surface protein

**Figure 4:**
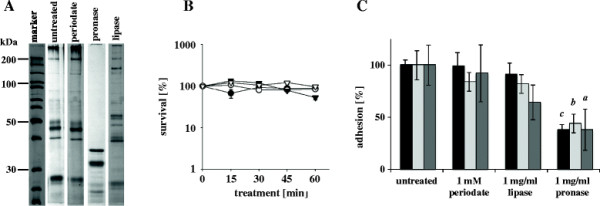
**Adhesion of treated*B. bifidum*S17 in comparison to untreated bacteria.** (**A**) SDS-PAGE of cell wall fractions of *B. bifidum* S17 after 30 min treatment with 1 mM periodate, 1 mg/ml lipase or 1 mg/ml pronase. 50 μg of protein were loaded in each lane. (**B**) Viability of untreated *B. bifidum* S17 (black circles) or during treatment with 1 mM pronase (white circles), 1 mg/ml lipase (black triangles) or 1 mg/ml periodate (white triangles). Samples were taken after 0 (pre-treatment control), 15, 30, 45, and 60 minutes and colony forming units were determined after growth on MRSC agar plates. (**C**) Adhesion of *B. bifidum* S17 treated for 30 min with either 1 mM periodate, 1 mg/ml lipase or 1 mg/ml pronase to T84 (black bars), Caco-2 (light grey bars) and HT29 (dark grey bars) cells. 10^8^ cfu/well of treated bacteria were added to IECs. Adherent bacteria were determined after growth on MRSC agar plates and expressed as percentage relative to untreated bacteria. Values are mean ± standard deviation of one representative of at least three independent experiments performed in triplicate measurements. Statistical analysis was performed by pairwise comparison to the respective positive control (i.e. adhesion of untreated *B. bifidum* S17) using Students *t*-test. Letters above bars indicate statistical significance (*a*: *p* < 0.05; *b*: *p* < 0.01; *c*: *p* < 0.001).

We hypothesized that BopA is involved in the strong adhesion of *B. bifidum* strains to IECs. To gain further insight into the chemical nature of the structures involved in adhesion, *B. bifidum* S17 was treated with 1 mM sodium periodate, 1 mg/ml lipase (type II from porcine pancreas) or 1 mg/ml pronase to digest or alter carbohydrates, lipids or proteins on the cell surface of *B. bifidum* S17. Preliminary experiments revealed that the protein pattern of the cell wall fraction is already significantly altered after 30 min of pronase or lipase treatment (Figure 4A) and bacteria remained viable during treatments for at least 60 min (Figure 4B). Thus, bacteria treated for 30 min were used in subsequent adhesion experiments.

Adhesion experiments with treated *B. bifidum* S17 to the three IEC lines T84, Caco-2 and HT29 showed significantly decreased adhesion after treatment with 1 mg/ml pronase (Figure 4C). In comparison to untreated *B. bifidum* S17, adhesion of pronase-treated bacteria to all cell lines tested was reduced to about 40%. Treatment of *B. bifidum* S17 with 1 mM periodate or 1 mg/ml lipase had no significant effect on adhesion for any of the tested IECs. Collectively these results suggest that a protein on the surface of intact bacteria of *B. bifidum* S17 is involved in adhesion to IECs.

### Adhesion of *B. bifidum* S17 in competition to purified BopA-His_6_

To further demonstrate that BopA is involved in adhesion of *B. bifidum* S17 to IECs, a C-terminal His_6_-fusion of BopA (BopA-His_6_) was expressed in *E. coli* BL21 (DE3) pMGS_P_BAD__*bopA*His_6_ (Table 1). Expression of BopA-His_6_ was induced in exponentially growing bacteria using 0.013 mM L-arabinose. Three hours after induction, bacteria were harvested and crude extracts were prepared.

**Table 1:** Bacterial strains and plasmids used in this study

**Strain**	**Relevant characteristics**	**Reference**
***E. coli***		
DH10B	Cloning host	Invitrogen™
BL21 (DE3)	Expression host	Invitrogen™
***Bifidobacterium***		
*B. bifidum* S17	intestinal isolate from a breast-fed infant	[23]
*B. bifidum* S16	intestinal isolate from a breast-fed infant	[23]
*B. bifidum* NCC189		NCC
*B. bifidum* NCIMB41171		NCIMB
*B. breve* S27	intestinal isolate from a breast-fed infant	This study
*B. breve* DSM20213	type strain, intestinal isolate from an infant	DSMZ
*B. longum/infantis* E18	intestinal isolate from an adult	[23]
*B. longum/infantis* NCC200		NCC
*B. longum* NCC2705	type strain	NCC
*B. longum* DSM20219	type strain, intestinal isolate from an adult	DSMZ
*B. adolescentis* NCC251	type strain	NCC
*B. adolescentis* DSM20083	type strain, intestinal isolate from an adult	DSMZ
*B. lactis* NCC362	type strain	NCC
*B. lactis* L15	isolate from a commercial dairy product	This study
*B. animalis* MB254	type strain	[23]
**Plasmids**		
pMDY23	*E. coli-Bifidobacterium* shuttle vector harbouring the *gusA* reporter gene, Spc^r^	[33]
pMGS_P_BAD__*bopA*His_6_	pMGS containing *bopA* under control of arabinose-inducible P_BAD_ promoter	This study
pMGS_P_gap__*bopA*His_6_	pMGS containing *bopA* under control of constitutive P_gap_ promoter	This study

**Figure 5:**
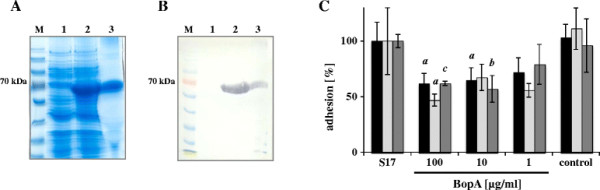
**Adhesion of*B. bifidum*S17 in competition to purified BopA-His**_**6**_. SDS-PAGE (**A**) and Western Blot (**B**) for detection of BopA-His_6_ in crude extract of *E. coli* BL21 (DE3) pMGS_*bopA*His_6_ before (lane 1) or after (lane 2) induction with 0.013 mM L-arabinose. Lane 3: purified BopA-His_6_ protein in pooled and dialysed fractions of the Ni-NTA-affinity chromatography. M: protein ladder. (**C**) Adhesion of *B. bifidum* S17 to T84 (black bars), Caco-2 (light grey bars) and HT29 (dark grey bars) cells after pre-incubation with 1, 10 or 100 μg/ml BopA-His_6_. As control IECs were incubated with 100 μg/ml BSA instead of BopA-His_6_. Adherent bacteria were determined after growth on MRSC agar plates. Results are shown as percent adherent bacteria relative to the positive control (untreated cells). Values are mean ± standard deviation of one representative of at least three independent experiments performed in triplicate measurements. Statistical analysis was performed by pairwise comparison to the respective positive control (i.e. adhesion of *B. bifidum* S17 without pre-incubation with protein) using Students *t*-test. Letters above bars indicate statistical significance (*a*: *p* < 0.05; *b*: *p* < 0.01; *c*: *p* < 0.001).

SDS-PAGE revealed a predominant band in crude extracts of bacteria induced with L-arabinose, which was absent in non-induced bacteria (Figure 5A). This band had a molecular mass of approximately 66 kDa, which is consistent with the predicted molecular mass of BopA-His_6_. Additionally, Western Blot using anti-His_6_ antibodies confirmed that the predominant band in the gel is a His-tagged protein of the expected size of BopA-His_6_ (Figure 5B).

For protein purification the cytoplasmic fraction of *E. coli* BL21 (DE3) pMGS_P_BAD__*bopA*His_6_ containing the majority of BopA-His_6_ as shown by SDS-PAGE (data not shown) was applied to the Ni-NTA column. Bound protein was eluted from the column by increasing concentrations of imidazole and collected in different fractions. A single peak of protein was detected by absorbance at 280 nm at a concentration of 40 mM imidazole (data not shown). All fractions collected during the peak of absorbance were pooled and dialysed. A single band of protein with a size of approximately 66 kDa corresponding to BopA-His_6_ protein was detected by SDS-PAGE and Western Blot in the pooled fractions (lanes 3 in Figure 5A and B).

For competitive adhesion assays, IECs were pre-incubated with 1, 10 or 100 μg/ml of purified BopA-His_6_ for 1 h prior to addition of intact bacteria of *B. bifidum* S17. 100 μg/ml of purified BopA-His_6_ significantly reduced adhesion of intact bacteria to all cell lines tested. Moreover, 10 μg/ml protein was sufficient to significantly reduce adhesion to T84 and HT29 cells (Figure 5C). Of note, as little as 1 μg/ml of purified BopA-His_6_ produced a similar reduction in adhesion of intact bacteria. However, these effects did not reach statistical significance. To exclude unspecific inhibition of adhesion, control experiments were performed in which cells were pre-incubated with 100 μg/ml BSA instead of purified BopA. This did not affect adhesion of *B. bifidum* S17 to IECs (Figure 5C).

In summary, adhesion of *B. bifidum* S17 to all IEC lines tested was significantly reduced after incubation of IECs with purified BopA-His_6_, clearly demonstrating that *B. bifidum* S17 competes with purified BopA-His_6_ for binding sites on IECs.

### Adhesion of bifidobacteria overexpressing *bopA*

**Figure 6:**
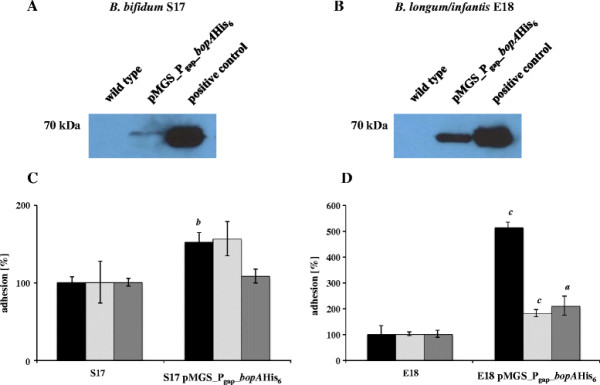
**Adhesion of bifidobacteria overexpressing*bopA***. His_6_-specific Western Blot on lysates of *B. bifidum* S17 (wt), *B. bifidum* S17 pMGS_P_gap__*bopA*His_6_ (**A**) or *B. longum/infantis* E18 (wt), and *B. longum/infantis* E18 pMGS_P_gap__*bopA*His_6_ (**B**) grown for 16 h. Lysates of *E. coli* DH10B pMGS_P_gap__*bopA*His_6_ were used as positive control for detection of BopA-His_6_. Adhesion of *B. bifidum* S17 pMGS_P_gap__*bopA*His_6_ (**C**) and *B. longum/infantis* E18 pMGS_P_gap__*bopA*His_6_ (**D**) to T84 (black bars), Caco-2 (light grey bars) and HT29 (dark grey bars) cells compared to their parental wildtype strains. Results are expressed as percent adherent bacteria relative to the initially added colony forming units. Values are mean ± standard deviation of one representative of at least three independent experiments performed in triplicate measurements. Statistical analysis was performed by pairwise comparison to the respective positive control (i.e. adhesion of *B. bifidum* S17 or *B. longum/infantis* E18 wildtype) using Students *t*-test. Letters above bars indicate statistical significance (*a*: *p* < 0.05; *b*: *p* < 0.01; *c*: *p* < 0.001).

In order to test if expression of *bopA* in bifidobacteria results in strains with improved adhesion characteristics, the recombinant strains *B. bifidum* S17 pMGS_P_gap__*bopA*His_6_ and *B. longum/infantis* E18 pMGS_P_gap__*bopA*His_6_ were generated. When these strains were cultured o/N in MRSC medium under standard conditions no additional protein bands were detected in total bacterial lysates by SDS-PAGE (data not shown). However, weak but consistent signals were detected by His_6_-specific Western Blot (Figure 6A and B) demonstrating successful expression of BopA-His_6_ in these strains.

Both strains were then compared to their wildtype parental strains in adhesion experiments. Adhesion of *B. bifidum* S17 pMGS_P_gap__*bopA*His_6_ to T84 cells was significantly increased to about 150% in comparison to *B. bifidum* S17 wildtype (Figure 6C). A similar increase in adhesion was observed when Caco-2 cells were used, however this effect was not significant. No improvement was observed for adhesion of *B. bifidum* S17 pMGS_P_gap__*bopA*His_6_ to HT29 cells. *B. longum/infantis* E18 is a strain that showed only very weak adhesion to the tested cell lines in previous experiments. Expression of *bopA* in this strain resulted in a highly increased adhesion to all IECs tested (T84: 511%; Caco-2: 180%; HT29: 209%) compared to *B. longum/infantis* E18 wildtype (Figure 6D).

In summary, the results of adhesion experiments with *B. bifidum* S17 and *B. longum/infantis* E18 strains expressing BopA-His_6_ further confirm the previous results and demonstrate that BopA plays an important role in adhesion to T84 and Caco-2 cells.

## Discussion

Adhesion of probiotic bacteria to IECs and mucus is thought to help these organisms to persist in the intestinal tract for prolonged periods of time, thus increasing the chances to exert their health-promoting effects. Furthermore, adhesion of probiotics to host cells could be an important prerequisite for the immunmodulatory effects of probiotics and was proposed to have a role in the competitive exclusion of pathogenic bacteria by competing for attachment sites [34-37]. As a consequence, adhesion to IECs is one of the criteria for the selection of probiotic strains for nutraceutical applications.

However, in healthy individuals, the luminal surface of the small and large intestinal epithelium is covered with a thick layer of secreted mucus consisting of two distinct layers: an inner layer firmly attached to the epithelium and a more loosely attached layer facing the intestinal lumen [38]. While bacteria are usually found to colonize the outer layer, the inner firmly attached layer is devoid of bacteria [39,40] thus questioning the importance of adhesion to IECs for activity and colonisation of probiotics under normal circumstances. However, under conditions of intestinal inflammation the mucus barrier is reduced and can be penetrated by bacteria [41-43]. Thus, under these conditions adhesion of probiotic bacteria to IECs might contribute to persistence of these organisms in the intestinal tract of the host for prolonged periods of time, thus increasing the chances to exert their health-promoting effects.

Here, we investigate the adhesive properties of *B. bifidum* S17, a strain with potent anti-inflammatory capacity both *in vitro* and *in vivo*[22,24], aiming to identify the components involved in adhesion to IECs and to improve the adhesive properties of this and other promising probiotic candidate strains. All analysed *B. bifidum* strains showed markedly better adhesion than other strains to all cell lines tested and all strains contained a *bopA* homolog, which is highly conserved among these strains. The three genes located upstream of *bopA* showed significant similarity to genes encoding the membrane permease subunits (*oppBC*) and an ATP-binding and -hydrolysing protein (*oppD*) of ABC transport systems. Downstream of *bopA*, a gene with high similarity to bacterial aminopeptidase C (*pepC*) was identified, which is transcribed in the opposite direction. An *oppA* homologue has been found in the *opp* operons of other *Bifidobacterium* genomes. However, the amino acid sequence of OppA of these strains shares no significant similarity with BopA.

There is a considerable amount of studies demonstrating lipoproteins as solute binding proteins of ABC transporters that mediate uptake of nutrients [44]. Moreover, lipoproteins of ABC transport systems are often associated with bacterial adhesion. For example, the lipoprotein P100 of *Mycoplasma hominis* is an OppA-like substrate-binding protein of a peptide transport system that has been characterised as multifunctional protein. In addition to cytoadherence P100 has been shown to mediate nutrient uptake and hydrolysis of extracellular ATP [45-47]. Similar results have been obtained with components of ABC transporters of *E. coliBrucella sp.* and *Streptococcus gordonii*[48-50].

Southern Blot analysis of genomic DNA of all *Bifidobacterium* strains tested for adhesion revealed a signal only for *B. bifidum* strains suggesting that only this species harbours homologues of *bop*A (Figure 3A). This data corroborates the observation of a previous study, which demonstrated the presence of BopA in eight *B. bifidum* strains but not in other *Bifidobacterium* strains as shown by PCR [20]. Northern blot analysis with the *B. bifidum* strains showed that *bopA* is expressed in these strains under conditions used to cultivate bacteria for adhesion assays. However, expression of *bopA* was variable amongst the strains and no apparent correlation between *bopA* mRNA levels as detected by Northern blot and adhesion could be observed.

To experimentally confirm these results, the involvement of BopA on adhesion of *B. bifidum* S17 was tested by enzymatically altering different surface components. Adhesion of intact bacteria was inhibited significantly by pre-treatment with pronase but treatment did not affect viability. These results strongly indicate that adhesion of *B. bifidum* S17 to IECs is, at least partially, mediated by proteinaceous structures of the cell surface, which would be the expected location for an adhesin.

Slight differences in the effects of treatments with pronase, lipase or periodate were observed between the cell lines used for experiments. Caco-2 and T84 cells differentiate to columnar epithelial cells and HT29 cells remain undifferentiated. Differential expression of receptor molecules on the surface might explain the differences between cell lines and cell fractions. Of note, a slight reduction in adhesion of intact bacteria was also observed for some cell lines after lipase treatment. These effects were only marginal and statistically not significant. Lipase treatment might alter the structure and composition of membrane lipids. Since BopA is a lipoprotein anchored to the cytoplasmatic membrane, alterations in the lipids of the membrane provide a possible explanation for the effects of lipase treatment.

To further investigate the role of BopA for adhesion of *B. bifidum* S17, a C-terminal His-tag fusion of the protein was purified after overexpression in *E. coli* BL21 (DE3). Adhesion of *B. bifidum* S17 to T84, Caco-2 and HT29 cells was significantly inhibited in competition to purified BopA protein, indicating that BopA interferes with the adhesion mechanism of *B. bifidum* S17 to IECs. Pre-incubation with higher concentration of purified BopA improved the statistical significance but did not increase the effect on adhesion of *B. bifidum* S17 to the tested cell lines. This indicates a saturation of receptor binding sites on the epithelial surface already at lower concentrations. This observation is in line with experimental data of adhesion studies with purified adhesion proteins of other organisms. For instance, the purified OmpA-like protein Lsa66 of *Leptospira interrogans* was shown to bind extracellular matrix (ECM) components specifically and saturable [51]. Furthermore, saturable fibronectin binding was shown for the CssA subunit of colonisation factor CS6 of enterotoxigenic *E. coli*[52] as well as for the *Campylobacter jejuni* adhesin FlpA [53].

The fact that inhibition of adhesion by purified BopA or after pre-treatment with pronase, lipase or periodate was incomplete points towards additional mechanisms of adhesion that are not affected by the treatments. The involvement of different adhesion mechanisms has been shown for the probiotic *L. rhamnosus* GG for which three different surface-localised components with a role in adhesion were identified [54]. Cooperative effects of different mechanisms of adhesion could represent a strategy of bifidobacteria (and other microorganisms) to increase their chances to successfully colonize the GIT. Two recent studies demonstrated that various *Bifidobacterium* strains express pili on their surface, which at least in one case are essential for host colonization [55,56]. Analysis of the genome of *B. bifidum* S17 revealed that the strain harbours three clusters of genes with high homology to pilus-coding genes of other bifidobacteria (data not shown). Several other genes encoding for proteins containing domains with known or suspected roles in adhesion to host tissues were identified in the genome of *B. bifidum* S17. Other studies indicate that surface hydrophobicity correlates with the adhesive capacity of bifidobacteria [8,57]. However, the actual relevance of the abovementioned factors to adhesion of *B. bifidum* S17 remain to be investigated experimentally.

To further corroborate the role of BopA for adhesion of *B. bifidum* S17, a vector for expression of *bopA* in bifidobacteria was constructed. In previous studies the P_*gap*_ promoter driving expression of *gap*, i.e. the gene encoding glycerinaldehyde-3-phosphate dehydrogenase, was successfully used for heterologous expression of proteins in bifidobacteria [33,58,59]. Thus, the vector pMGS_P_gap__*bopA*His_6_ was constructed for P_gap_-driven expression of BopA-His_6_ in bifidobacteria. This vector was successfully transformed into *B. bifidum* S17 and *B. longum/infantis* E18. Unfortunately, no additional protein band corresponding to BopA-His_6_ could be detected in crude extracts of the recombinant strains by SDS-PAGE (data not shown). Detection of proteins expressed in bifidobacteria by SDS-PAGE seems to be difficult since to the best of our knowledge no studies showing overexpression in bifidobacteria by SDS-PAGE are available. In those studies that provide protein data, successful expression of proteins in bifidobacteria is demonstrated by Western Blot analysis [59,60]. Indeed, Western Blot analysis using a His_6_-specific antibody confirmed the presence of BopA-His_6_ both in *B. bifidum* S17 pMGS_P_gap__*bopA*His_6_ and *B. longum/infantis* E18 pMGS_P_gap__*bopA*His_6_.

These strains were then used in adhesion experiments and compared to their parental wildtype strains. Overexpression of *bopA* in *B. bifidum* S17 significantly increased adhesion to IECs. More importantly, expression of *bopA* in *B. longum*/*infantis* E18, a strain that does not encode for a BopA homologue as shown by Southern Blot (Figure 3A) and displays extremely weak adhesion to IECs (Figure 1), led to a dramatic increase in adhesion to all cell lines tested. Overall, the effect on adhesion with *B. longum/infantis* E18 overexpressing *bopA* was by far more prominent than with *B. bifidum* S17 pMGS_P_gap__*bopA*His_6_. This can be explained with the fact that the adhesin BopA is not present in *B. longum/infantis* E18 wildtype. In contrast, expression of BopA in *B. bifidum* S17 enhanced adhesion to IECs to a lesser extent, because BopA-His_6_ is expressed additional to wildtype BopA in this strain.

## Conclusions

Collectively, these results identify BopA as an adhesin mediating adhesion of *B. bifidum* strains to IECs. Furthermore, increased adhesion of *B. bifidum* S17 and *B. longum/infantis* E18 following enhanced expression of *bopA* is one of the first reports on improved probiotic characteristics of *Bifidobacterium* strains. Although the use of genetically modified organisms in functional (probiotic) food products is currently not accepted in the European markets, the tools generated in the present study are suitable to generate recombinant bifidobacteria with optimised probiotic characteristics. To our knowledge, our study is the first report on enhanced probiotic properties by expression of a bifidobacterial adhesin in strains of *Bifidobacterium*. The only other study so far has shown prolonged colonisation of a *B. breve* strain expressing a gene involved in bile resistance of *Listeria monocytogenes*[61]. Besides this, only a limited number of studies demonstrate improved probiotic properties of recombinant lactic acid bacteria. One example is a recombinant *Lactobacillus paracasei* strain expressing the gene coding for the *Listeria* adhesion protein (Lap), which was shown to protect Caco-2 cells from infection with *Listeria monocytogenes* by interaction with host cell receptor Hsp60 [62]. In further studies, the relevance of BopA for *in vivo* colonisation will be investigated. Furthermore, additional factors that are involved in adhesion of *B. bifidum* S17 need to be identified to completely understand the mechanisms of adhesion of this promising anti-inflammatory probiotic organism.

## Materials and methods

### Bacterial strains and culture conditions

Bifidobacteria (Table 1) were grown in Lactobacilli MRS medium (Difco) supplemented with 0.5 g/l L-cysteine (MRSC) for 16 h to stationary phase at 37 °C under anaerobic conditions. In addition, *E. coli* strains (Table 1) DH10B and BL21 (DE3) were used for cloning and protein expression, respectively. *E. coli* was cultivated in 2× Trypton Yeast (TY) medium and grown to stationary phase at 37 °C with agitation. For cultivation of strains harbouring plasmids, the respective medium was supplemented with 100 μg/ml spectinomycin.

### Culture conditions of intestinal epithelial cells

Eukaryotic cell lines T84 (ATCC), Caco-2 (ATCC) and HT29 (DSMZ) were maintained in DMEM/Ham’s F12 or DMEM medium, respectively. Medium for T84 cells was supplemented with 10% (v/v) heat-inactivated foetal calf serum (FCS), and 1% (v/v) penicillin-streptomycin solution. Medium for Caco-2 cells was supplemented with 10% (v/v) FCS, 1% (v/v) non-essential amino acids (NEAA), and 1% (v/v) penicillin-streptomycin solution and for HT29 cells with 5% (v/v) FCS, 1% (v/v) NEAA, and 1% (v/v) penicillin-streptomycin solution. Cells were incubated in cell culture incubators at 37 °C with 5% CO_2_. Medium was changed every two to three days and cells were subcultured according to supplier’s guidelines. For experiments, cells were grown for 9–11 (T84) or 18–21 (Caco-2) days to fully differentiated monolayers. HT29 cells were used as undifferentiated monolayers at 90–95% confluence, i.e. approximately five days after seeding. At this stage about 1 × 10^8^ cells were counted per well for all cell lines.

### Adhesion assay

Prior to incubation with bacteria, normal medium of the cells was replaced with cell culture medium without serum and antibiotics. For adhesion assays, bacteria were grown for 16 h under appropriate conditions and washed once in phosphate buffered saline (PBS; pH 7.4) and adjusted to an OD_600_ corresponding to 1 × 10^9^ colony forming units (cfu) per ml in PBS. 100 μl (or 1 × 10^8^ cfu) of this bacterial suspension were added to each well. After incubation for 1 h at 37 °C non-adherent bacteria were removed by washing cells three times with PBS. Cell monolayers were then lysed by the addition of ice-cold bidistilled H_2_O and serial ten-fold dilutions in PBS were plated in spots of 10 μl on MRSC-agar plates to enumerate cfu of adherent bacteria. Adhesion of the bacteria to the eukaryotic cells was then calculated as percentage of the number of bacteria initially added to the wells, which was determined by spot plating of the bacterial suspension added to the monolayers. All adhesion experiments were performed at least in three independent biological replicates, i.e. bacteria of three independent bacterial cultures were tested on cells of different passages.

To test adhesion of bifidobacteria in competition purified protein experiments were carried out as described above using monolayers that were pre-treated for 1 h with purified BopA (100 μg, 10 μg or 1 μg total protein as indicated). In these experiments, adhesion of the bacteria to the eukaryotic cells was calculated as percentage of adherent bacteria relative to the positive control (no purified BopA protein).

### Treatment of *B. bifidum* S17 and viability test

To digest proteins, carbohydrates or lipids on the cell surface of *B. bifidum* S17, bacteria were grown under standard conditions. After centrifugation (10 min, 5,000 × g) bacteria were washed once with PBS and adjusted to an OD_600_ corresponding to 1 × 10^9^ cfu/ml and treated with lipase (1 mg/ml in PBS), pronase (1 mg/ml in pronase buffer: 10 mM sodium acetate, 5 mM potassium acetate, pH 7.5) or periodate (1 mM in 1 M glycine). Untreated and periodate-treated bacteria were incubated in the dark at RT with agitation whereas pronase- and lipase-treated bacteria were incubated at 37 °C in a water bath. Treatments were maintained for up to 60 min as indicated in the Results section. For adhesion experiments cells were treated for 30 min, then centrifuged (10 min, 14,000 × g) and washed once with PBS. To apply treated bacteria to adhesion assays, bacteria were pelleted by centrifugation and OD_600_ was re-adjusted to obtain 1 × 10^8^ cfu/ml. Adhesion of treated bacteria was tested as described and calculated as percentage of adherent bacteria relative to the positive control (untreated bacteria), which was set to 100%.

To check viability of bacteria during and after the treatment, samples were taken after 0, 15, 30, 45 and 60 min and serial dilutions were plated on MRSC agar plates to determine cfu counts. Furthermore, cell wall fractions of bacteria were prepared of samples at different time points during the treatments as by pelleting bacteria and resuspension in lysis buffer (1M sucrose, 11.4 mM PMSF, and 15 mg/ml lysozyme in 50 mM Tris-HCl; pH 7.6). After incubation for 90 min at 37 °C protoplasts were pelleted by three centrifugations at 1,500 xg for 1 min and the supernatant was retained representing the cell wall fraction.

### Southern blot

Southern Blot was performed according to a standard protocol [63] using *Eco*RI-digested chromosomal DNA of *Bifidobacterium* strains (Table 1) separated on a 0.8% (w/v) agarose gel and blotted on a nylon membrane (GE Healthcare). For detection of *bopA* homologues, a digoxygenin-11-dUTP-labelled probe was amplified by PCR using chromosomal DNA of *B. bifidum* S17 as template and the oligonucleotides *bopA*_f_SB (5’-CTCGTGCTGTTCAGGAGAGG-3’) and *bopA*_r_SB (5’-TCACTTCTCCCAGCCGAGGTTC-3’). Detection of the hybridised probes was performed using an anti-digoxygenin antibody coupled to the enzyme alkaline phosphatase (Cat. #11 093 274 910, Roche) and visualised on an X-ray film using a commercial CSPD substrate (Roche).

### Northern blot

For Northern Blot, RNA was isolated using a standard protocol [63] from *B. bifidum* strains (Table 1) after growth for 16 h under standard conditions. Briefly, bacteria were mixed with Quenching buffer (60% (v/v) methanol, 66.7 mM HEPES, pH 6.5, -40 °C) and harvested by centrifugation (4,000 × g, 20 min, 4 °C). The pellet was resuspended in 200 μl ice-cold H_2_O and mixed with 400 μl phenol (pH 4.5-5), 100 μl chloroform, 30 μl 10% (w/v) SDS, 30 μl sodium acetate (3 M, pH 5.2) and 250 mg glass beads to disrupt bacteria in a RiboLyser (3 cycles, each for 40 seconds at 4 °C) set to full speed. Cell debris, glass beads and phenol/chloroform were removed by centrifugation (15 min, 14,000 × g, 4 °C). The aqueous phase containing RNA was mixed with 1.5 volumes of 99% ethanol and incubated o/N at −20 °C. After centrifugation (14,000 × g, 15 min, 4 °C) the pellet was washed once with ice-cold 70% (v/v) ethanol and air-dried. The dry pellet was resuspended in H_2_O and RNA concentration was determined by measuring absorbance at 260 nm.

Residual DNA was removed by a treatment with DNaseI, which was repeated until no DNA was left in the RNA sample, which was checked by PCR using primers specific for 16 S rDNA (16S_fD1: 5’-AGAGTTTGATCCTGGCTCAG-3’ and 16S_rP2: 5’-ACGGCTACCTTGTTACGACTT-3’, [64]) and RNA sample as template. Following the DNaseI treatment RNA samples were purified using the RNeasy Mini Kit (Qiagen) according to manufacturer’s instructions.

DNA-free RNA was separated on a 0.8% (w/v) agarose gel supplemented with 10% (v/v) formaldehyde (37%) and blotted onto a nylon membrane (GE Healthcare). A digoxygenin-11-dUTP-labelled DNA probe complementary to the target RNA molecule was amplified by PCR using chromosomal DNA of *B. bifidum* S17 as template and the oligonucleotides *bopA*_f_NB (5’-AAGGTCCAGAGCGGCAAGAG-3’) and *bopA*_r_NB (5’-CACACCGGAGTCGTAGGAAC-3’), hybridised to the membrane and detected as described above.

### Purification of the *B. bifidum* S17 BopA protein

For expression of *bopA* in *E. coli* BL21 (DE3), a vector was constructed based on the previously published reporter plasmid pMDY23 ([33], Table 1). The *gusA* gene encoded on pMDY23 was removed together with a range of restriction sites by amplification of the pMDY23 vector backbone using the oligonucleotides *gusA*_f_*Fsp*I (5’-TGCGCAGCCTCAGCCTGCGGAACGCGC-3’, *Fsp*I site underlined) and *gusA*_r_*Fsp*I (5’-TGCGCAGCTGTAGACCAAGTTTACTC-3’, *Fsp*I site underlined). The vector backbone was then digested with *Fsp*I and ligated to the multiple cloning site of pBluescript, which was amplified using the oligonucleotides T3 (5’-AATTAACCCTCACTAAAGGG-3’) and T7 (5’-TAATACGACTCACTATAGGG-3’), resulting in the *E. coli-Bifidobacterium* shuttle vector pMGS. For arabinose-inducible expression, the araC gene and P_BAD_ were amplified from pREDI [65] using the oligonucleotides pBAD_f_SacII (5’-CATCATCCGCGGTTATTATGACAACTTGACG-3’, *Sac*II site is underlined) and pBAD_r_SpeI (5’-CATCATACTAGTACCCTCCTTAGAGCTCG-3’, *Spe*I site is underlined). The PCR product was digested using restriction enzymes *Sac*II and *Spe*I and ligated to the *Spe*I/*Sac*II-digested pMGS plasmid resulting in the vector pMGS_P_BAD_. The sequence of *bopA* was amplified by PCR using chromosomal DNA of *B. bifidum* S17 and the oligonucleotides *bopA*_f_*Spe*I (5’-GGACTAGTATGAGTTTTGCATCTACCGC-3’, *Spe*I site is underlined) and *bopA*His_r_*Hind*III (5’-CCCAAGCTTTCAGTGGTGGTGGTGGTGGTGCTTCTCCCAGCCGAGGTTC-3’, *Hind*III site is underlined). The *Spe*I- and *Hind*III-digested PCR fragment was ligated to the *Spe*I/*Hind*III cut plasmid pMGS_P_BAD_ and the resulting plasmid (pMGS_P_BAD__*bopA*His_6_) was transformed in *E. coli* BL21 (DE3). For production of recombinant BopA-His_6_*E. coli* BL21 (DE3) pMGS_P_BAD__*bopA*His_6_ was cultivated in 2× TY medium supplemented with 100 μg/ml spectinomycin, under standard conditions until an OD_600_ of 0.5 was reached. Expression of *bopA* was induced by addition of 0.013 mM L-arabinose and bacteria were grown for further 3 h at 37 °C with agitation to accumulate recombinant BopA. Then bacteria were harvested by centrifugation (4 °C, 5,000 × g, 10 min), and washed once with binding buffer (30 mM Tris, 500 mM NaCl, 5 mM imidazole, 30% (v/v) glycerol, pH 7.9). Bacterial cells were disrupted using a French Press at a pressure of 850 PSI. The cytoplasmic fraction was obtained from the lysate by centrifugation at 4 °C, 250,000 × g for 1.5 h in an ultracentrifuge. For purification of BopA-His_6_ by Ni-NTA affinity chromatography, the cytoplasmic fraction was loaded on a 1 ml HisTrap FF column (GE Healthcare) fitted to an ÄKTA FPLC purifier system (GE Healthcare), which was calibrated prior to loading with elution buffer (30 mM Tris, 500 mM NaCl, 500 mM imidazole, 30% (v/v) glycerol, pH 7.9) and then with binding buffer. Bound protein was eluted by a linear imidazole gradient (5 mM to 500 mM). The eluate was collected and respective fractions were run on a SDS-PAGE to check for size and purity of the recombinant protein. Pure fractions showing a signal of the expected size were pooled and subsequently dialysed against 20% (v/v) glycerol solution (o/N, 4 °C) to remove imidazole from the buffer. After dialysis, purified protein was concentrated by incubation of dialysis tubes in polyethylene glycol (PEG 20.000) for 2–3 h. To apply a defined protein amount to adhesion assays, protein concentration was determined using a BCA protein assay (Thermo Scientific).

### Western blot

For Western Blot analysis, equivalent amounts of protein as determined by BCA protein assay were separated by SDS-PAGE and subsequently electroblotted onto nitrocellulose membrane. After blocking of unspecific protein binding with blocking buffer (5% milk powder in PBS), nitrocellulose membrane was incubated with anti-His_6_ antibody (1:1,000 in PBS; Cat. #11922416001, Roche) for 1 h at room temperature. After three washings with PBS, the membrane was incubated with a rabbit-anti-mouse horseradish peroxidase-coupled secondary antibody (1:10,000 in blocking buffer; Cat. #31430, Thermo Scientific) for 1 h at room temperature. Detection of horseradish peroxidase was performed using ECL Western Blotting substrate (Thermo Scientific) and chemiluminescence was detected on X-ray films.

### Expression of *bopA* in bifidobacteria

For expression of *bopA* in *B. bifidum* S17 and *B. longum/infantis* E18 the promoter sequence of glyceraldehyde-3-phosphate dehydrogenase gene (BBIF_0612, P_gap_) was amplified using chromosomal DNA of *B. bifidum* S17 and the oligonucleotides P_gap__f_*Bgl*II (5’-GAAGATCTGCGGAATGCCTCGCATCGAATC-3’, *Bgl*II site underlined) and P_gap__r_*Sal*I (5’-GACGACGTCGACCTCCCTTTGTAGGGTAGA-3’, *Sal*I site underlined) and ligated in the *Bgl*II and *Sal*I-cut plasmid pMGS yielding pMGS_P_gap_. The coding sequence of *bopA* was amplified by PCR using chromosomal DNA of *B. bifidum* S17 and the oligonucleotides *bopA*_f_*Sal*I (5’-GACGTAGTCGACATGAGTTTTGCATCTACCGC-‘3, *Sal*I site underlined) and *bopA*His_r_*Hind*III (5’-CCCAAGCTTTCAGTGGTGGTGGTGGTGGTGCTTCTCCCAGCCGAGGTTC-3’, *Hind*III site underlined) and the *Sal*I- and *Hind*III-digested PCR fragment was ligated to the *Sal*I/*Hind*III cut plasmid pMGS_P_gap_. The resulting plasmid (pMGS_P_gap__*bopA*His_6_) was transformed in *B. bifidum* S17 and *B. longum/infantis* E18 to test adhesion with constitutively overexpressed *bopA.*

## Abbreviations

ATCC, American type culture collection; DMEM/Ham’s F12, Dulbecco`s modified eagle medium and Ham’s F12 nutrient mixture; DMEM, Dulbecco’s modified eagle medium; DSMZ, German collection for microorganisms and cell culture; FCS, Foetal calf serum; GIT, Gastrointestinal tract; IEC, Intestinal epithelial cell; NCBI, National center for biotechnology information; NCC, Nestlé culture collection; NEAA, Non-essential amino acid; PBS, Phosphate buffered saline; PSI, Pound-force per square inch.

## Competing interests

The authors declare that they have no competing interests.

## Authors’ contributions

MG, DZ and CU Riedel designed experiments. MG, DZ and VG performed experiments. MG, DZ, JY and CU Riedel analysed data. MG, JY and CU Riedel wrote the manuscript. All authors read and approved the final manuscript.

## Supplementary Material

Additional file 1 **Table SA1.** Statistical analysis of the difference in adhesion of *B. bifidum* strains to all other strains used in this study. Click here for file

Additional file 2 **Table SA2.** BLAST analysis of the BopA of *B. bifidum* MIMBb75. Click here for file

Additional file 3 **Table SA3.** Annotation of BopA locus of *B. bifidum* strains S17, NCIMB41171, PRL2010 and MIMBb75. Click here for file

Additional file 4 **Figure S1.** Alignment of BopA sequences of *B. bifidum* S17, NCIMB41171, PRL2010 and MIMBb75. Click here for file
