# Supplementary material for: Improved adhesive properties of recombinant bifidobacteria expressing the Bifidobacterium bifidum-specific lipoprotein BopA
Source: Microb Cell Fact. 2012 Jun 13;11:80. doi: 10.1186/1475-2859-11-80 (PMC3408352; doi:10.1186/1475-2859-11-80)
Supplement: Additional file 1 — Table SA1. Statistical analysis of the difference in adhesion of B. bifidum strains to all other strains used in this study. [file 1475-2859-11-80-S1.pdf]

Table A1: Statistical analysis of the difference in adhesion of *B. bifidum* strains to all other strains used in this study. Statistical analysis was performed by pairwise comparison of the adhesion values of each of the four *B. bifidum* strains (S16, S17, NCC189 and NCIMB41171) to each of the other strains (*B. breve* strains S27 and DSM20213, *B. longum/infantis* strains E18 and NCC200, *B. adolescentis* strains NCC251 and DSM20083, *B. lactis* strains NCC362 and L15, *B. animalis* MB254, and *B. longum* strains NCC2705 and DSM20219) using Student's t-test. For each comparison the *p*-values is indicated and the difference was considered statistically significant if $p < 0.05$.

**Cell line**		**T84**				**Caco-2**				**HT29**			
B. bifidum		**S16**	**S17**	**NCC 189**	**NCIMB 41171**	**S16**	**S17**	**NCC 189**	**NCIMB 41171**	**S16**	**S17**	**NCC 189**	**NCIMB 41171**
B. breve	**S27**	< 0.001	< 0.001	< 0.001	< 0.001	0.002	< 0.001	< 0.001	< 0.001	0.001	< 0.001	0.004	< 0.001
B. breve	**DSM20213**	0.001	< 0.001	< 0.001	< 0.001	0.002	< 0.001	< 0.001	< 0.001	0.001	< 0.001	0.005	< 0.001
B. longum ssp. infantis	**E18**	< 0.001	< 0.001	< 0.001	< 0.001	0.004	< 0.001	< 0.001	< 0.001	0.001	< 0.001	0.004	< 0.001
B. longum ssp. infantis	**NCC200**	0.001	< 0.001	< 0.001	< 0.001	0.002	< 0.001	< 0.001	< 0.001	0.002	< 0.001	0.010	0.001
B. adolescentis	**NCC251**	0.011	< 0.001	< 0.001	< 0.001	0.038	< 0.001	0.001	< 0.001	0.003	< 0.001	0.012	0.002
B. adolescentis	**DSM20083**	0.030	0.004	< 0.001	0.001	0.017	< 0.001	< 0.001	< 0.001	0.002	< 0.001	0.007	< 0.001
B. lactis	**NCC362**	0.002	< 0.001	< 0.001	< 0.001	0.003	< 0.001	< 0.001	< 0.001	0.002	< 0.001	0.007	< 0.001
B. lactis	**L15**	0.002	< 0.001	< 0.001	< 0.001	0.016	< 0.001	< 0.001	< 0.001	0.002	< 0.001	0.009	0.001
B. animalis	**MB254**	0.001	< 0.001	< 0.001	< 0.001	0.010	< 0.001	< 0.001	< 0.001	0.005	< 0.001	0.024	0.005
B. longum	**NCC2705**	0.001	< 0.001	< 0.001	< 0.001	0.007	< 0.001	< 0.001	< 0.001	0.002	< 0.001	0.010	0.002
B. longum	**DSM20219**	0.001	< 0.001	< 0.001	< 0.001	0.002	< 0.001	< 0.001	< 0.001	0.001	< 0.001	0.004	< 0.001
