# Supplementary material for: Improved adhesive properties of recombinant bifidobacteria expressing the Bifidobacterium bifidum-specific lipoprotein BopA
Source: Microb Cell Fact. 2012 Jun 13;11:80. doi: 10.1186/1475-2859-11-80 (PMC3408352; doi:10.1186/1475-2859-11-80)
Supplement: Additional file 2 — Table SA2. BLAST analysis of the BopA of B. bifidum MIMBb75. [file 1475-2859-11-80-S2.pdf]

Table A2: BLAST analysis of the BopA of *B. bifidum* MIMBb75. The amino acid sequence of BopA of B. bifidum MIMBb75 was run against the on-redundant protein sequences using blastp. Annotation, E-value, coverage and identity of the hit with the highest homology to BopA in each of the 32 fully sequenced bifidobacterial genomes is shown.

Strain	Annotation	E-value	Coverage [%]	Identity [%]
Bifidobacterium bifidum				
PRL2010	oligopeptide-binding protein OppA	0	100	99
S17	peptide/nickel ABC transporter, extracellular solute-binding protein	0	100	99
NCIMB41171	extracellular solute binding protein	0	96	98
Bifidobacterium animalis subsp. lactis				
BB-12	unnamed protein product	4e-04	38	22
BLC1	unnamed protein product	8e-05	38	22
Bl-04	ABC transporter substrate-binding protein	6e-05	38	22
DSM10140	dipeptide ABC transporter periplasmic protein	3e-05	28	24
V9	unnamed protein product	3e-05	28	24
AD011	protein DppA	0.26	35	22
Bifidobacterium adolescentis				
ATCC15703	peptides ABC transporter substrate-binding protein	4e-08	66	22
L2-32	hypothetical protein BIFADO_00773	5e-08	66	22
Bifidobacterium breve				
UCC2003	oligopeptide-binding protein oppA	8e-154	93	44
DSM20213	putative extracellular solute-binding dependent transport lipoprotein	7e-28	24	41
ACS-071-V-Sch8b	unnamed protein product	3e-153	93	44

Table A2 (continued): BLAST analysis of the BopA of *B. bifidum* MIMBb75.

Strain	Annotation	E-value	Coverage [%]	Identity [%]
Bifidobacterium longum subsp. infantis				
157F	peptides ABC transporter substrate-binding protein	1e-05	28	28
ATCC15697	extracellular solute-binding protein, family 5	4e-155	93	44
Bifidobacterium longum subsp. longum				
NCC2705	DppA2	0.008	40	65
DJO10A	COG4166: ABC-type oligopeptide transport system, periplasmic component	0.17	20	41
BBMN68	oppa1	0.022	81	24
F8	ABC-type dipeptide transport system, periplasmic component	2e-05	15	34
JCM1217	dipeptide ABC transporter substrate-binding protein	0.002	61	24
JDM301	family 5 extracellular solute-binding protein	8e-155	93	44
KACC91563	unnamed protein product	0.003	59	43
Bifidobacterium dentium				
Bd1	sugars ABC transporter substrate-binding protein	0.074	5	50
ATCC27678	hypothetical protein BIFDEN_00090	1e-09	46	26
ATCC27679	dipeptide ABC superfamily ATP binding cassette transporter, binding protein	1e-09	46	26
JCVIHMP022	ABC transporter, substrate-binding protein, family 5	2e-10	46	26

Table A2 (continued): BLAST analysis of the BopA of *B. bifidum* MIMBb75.

Strain	Annotation	E-value	Coverage [%]	Identity [%]
B. angulatum				
DSM20098	hypothetical protein BIFANG_02491	3e-09	45	26
B. pseudocatenulatum				
DSM20438	hypothetical protein BIFPSEUDO_02658	9e-19	72	25
B. catenulatum				
DSM16992	hypothetical protein BIFCAT_01573	2e-18	72	25
B. gallicum				
DSM20093	bacterial extracellular solute-binding protein, family 5	2e-08	72	25
