# Supplementary material for: Improved adhesive properties of recombinant bifidobacteria expressing the Bifidobacterium bifidum-specific lipoprotein BopA
Source: Microb Cell Fact. 2012 Jun 13;11:80. doi: 10.1186/1475-2859-11-80 (PMC3408352; doi:10.1186/1475-2859-11-80)
Supplement: Additional file 3 — Table SA3. Annotation of BopA locus of B. bifidum strains S17, NCIMB41171, PRL2010 and MIMBb75. [file 1475-2859-11-80-S3.pdf]

Table A3: Annotation of BopA locus of *B. bifidum* strains S17, NCIMB41171, PRL2010 and MIMBb75.

Gene		Size [bp]	amino acids	% GC	Annotated function
***B. bifidum* S17**					
oppB	BBIF_0633	984	327	60.26	peptide/nickel transport system, permease protein
oppC	BBIF_0634	945	314	62.64	peptide/nickel transport system, permease protein
oppD	BBIF_0635	2199	732	64.93	peptide/nickel transport system, ATP-binding protein
bopA	BBIF_0636	1782	593	61.16	peptide/nickel transport system, extracellular solute-binding protein
pepC	BBIF_0637	1419	472	62.93	aminopeptidase C
***B. bifidum* NCIMB41171**					
oppB	4881-5864	984	327	60.26	contig 2.9, protein not annotated
oppC	5886-6830	945	314	62.64	contig 2.9, protein not annotated
oppD	6848-9046	2199	732	64.84	contig 2.9, protein not annotated
bopA	9210-11000	1782	593	61.22	extracellular solute binding protein
pepC	12614-11196C	1418	472	64.64	contig 2.9, protein not annotated
***B. bifidum* PRL2010**					
oppB	BBPR_0609	984	327	60.26	oligopeptide transport system permease protein
oppC	BBPR_0610	945	314	62.64	oligopeptide transport system permease protein
oppD	BBPR_0611	2199	732	64.89	oligopeptide transport system ATP-binding protein
bopA	BBPR_0612	1782	593	61.22	oligopeptide binding protein (oppA)
pepC	BBPR_0613	1377	458	62.74	aminopeptidase C
***B. bifidum* MIMBb75**					
oppD	1-933	933	310	63.98	putative ATPase
bopA	1106-2887	1782	593	61.16	putative cell surface lipoprotein
pepC	3419-3084C	336	111	63.69	putative aminopeptidase C
