# Supplementary material for: Improved adhesive properties of recombinant bifidobacteria expressing the Bifidobacterium bifidum-specific lipoprotein BopA
Source: Microb Cell Fact. 2012 Jun 13;11:80. doi: 10.1186/1475-2859-11-80 (PMC3408352; doi:10.1186/1475-2859-11-80)
Supplement: Additional file 4 — Figure S1. Alignment of BopA sequences of B. bifidum S17, NCIMB41171, PRL2010 and MIMBb75. [file 1475-2859-11-80-S4.pdf]

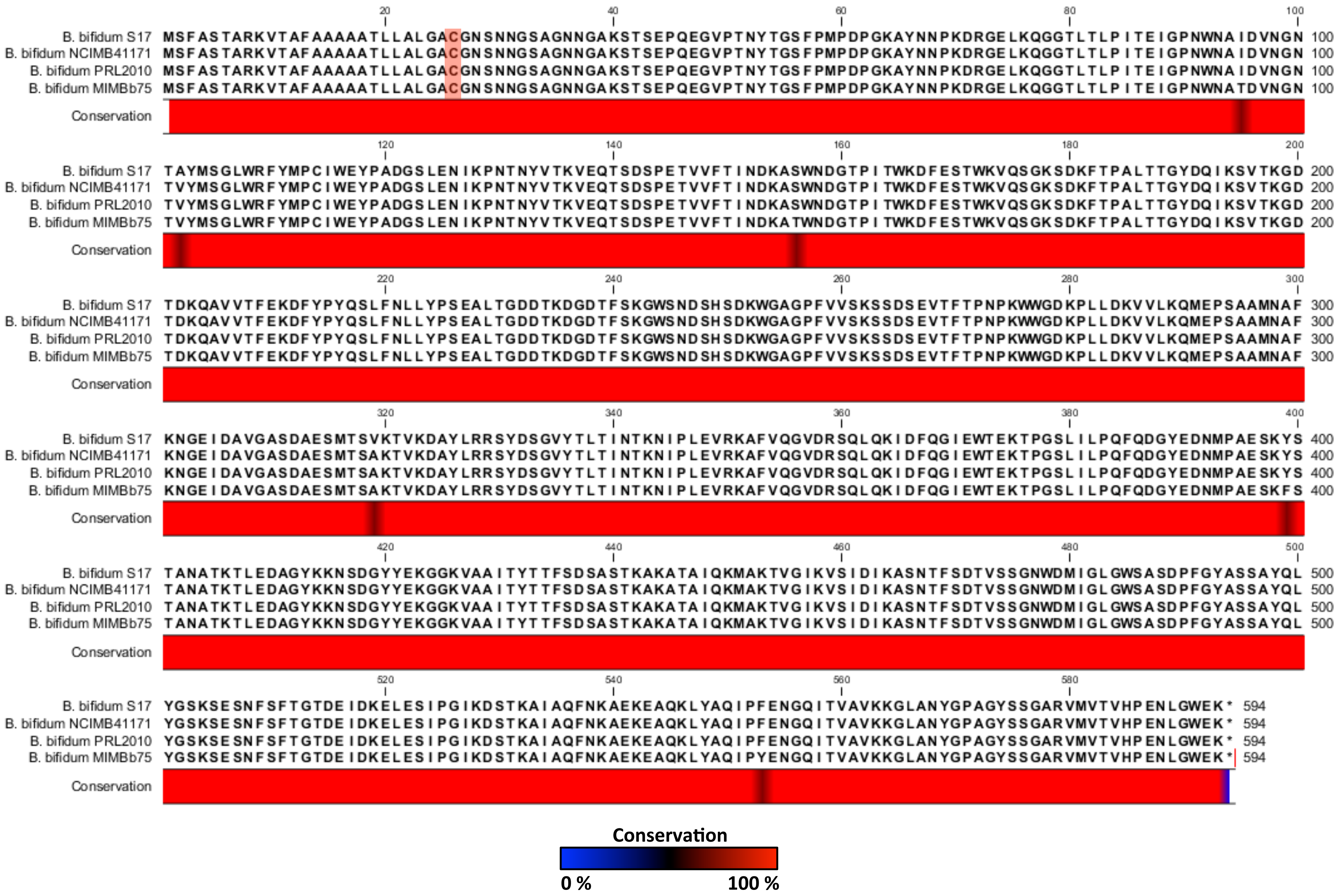

Figure S1: Alignment of BopA sequences of *B. bifidum* S17, NCIMB41171, PRL2010 and MIMBb75. Conservation of the amino acid sequence is indicated by a blue-to-red colour scheme (blue low conservation; red high conservation). The cysteine residue of the lipobox motif at position 26, which is important for thioacylation, is highlighted with a red box.
